# Recycling Pomelo Peel Waste in the Form of Hydrochar Obtained by Microwave-Assisted Hydrothermal Carbonization

**DOI:** 10.3390/ma15249055

**Published:** 2022-12-18

**Authors:** Yu-Jie Wang, Nan Li, Guo-Rong Ni, Chun-Huo Zhou, Xin Yin, Hua-Jun Huang

**Affiliations:** Key Laboratory of Agricultural Resource and Ecology in the Poyang Lake Basin of Jiangxi Province, School of Land Resources and Environment, Jiangxi Agricultural University, Nanchang 330045, China

**Keywords:** pomelo peel, hydrothermal carbonization, microwave, calcium oxide, hydrochar

## Abstract

Pomelo peel (PP) is a kind of solid waste that is produced in the processing industry of honey pomelo. This study deeply explored the feasibility of recycling PP in the form of hydrochar (HC) by microwave-assisted hydrothermal carbonization (HTC) technology. Under the non-catalytic reaction conditions, the yield of hydrochar initially increased with the rise of reaction temperature (150–210 °C) until it remained relatively stable after 210 °C. Under the CaO-catalytical reaction condition, the yield of hydrochar did not change much at first (150–190 °C) but decreased significantly when the reaction temperature exceeded 190 °C. After the microwave-assisted HTC treatment, the PP-derived HC presented higher aromaticity, carbonization degree, porosity, and caloric value. Compared with raw PP, the nutrients in HC were more stable (conducive to being used as slow-release fertilizer). The application of CaO increased the pH value of HC and effectively promoted the accumulation of phosphorus in HC. The HC produced at 210 °C without any catalyst possessing a high devolatilization ability. Additionally, the HC obtained at 190 °C with CaO as the catalyst presented a high combustion property. In general, PP-derived HC showed great application potential in the field of soil remediation/improvement and solid fuels. This preliminary study would undoubtedly provide some important fundamental understanding of the microwave-assisted HTC of PP.

## 1. Introduction

In the process of people’s consumption of pomelo, a by-product, pomelo peel (PP), will inevitably be produced, with a global production of 2.8–4.7 million tons, and most of it will be treated as waste. PP is rich in various nutrients and functional compounds, and it is a potential resource that can be used [1]. Through the proper treatment of PP, many high-value-added products or ingredients can be produced, and, at the same time, environmental pollution can be reduced. At present, there are three main ways to utilize PP: first, it is directly transformed into food materials (honey PP, tea, jam, etc.); second, extracting functional components (essential oil, pectin, polyphenols, etc.) from PP that can be used in food, pharmaceutical, chemical, and other fields; and third, PP can be converted into functional carbon materials or bioenergy [1,2,3,4].

In the field of functional carbon materials, PP has been exploited in many ways. In particular, it can be utilized as a material to fabricate adsorbents for the elimination of aqueous/gaseous pollutants (such as heavy metals, dyes, oils, and H_2_S) [5,6,7,8], catalysts for the degradation of organic pollutants [9,10,11,12], electrodes for preferential removal/recovery of heavy metals from contaminated water and high-performance supercapacitors [13,14,15]. The involved processes for the preparation of functional carbon materials include pyrolysis, hydrothermal carbonization (HTC), H_2_SO_4_ carbonization, annealing, physical/chemical activation, and so on. PP is rich in monosaccharides (glucose), disaccharides (fructose, sucrose), polysaccharides (cellulose, hemicellulose, pectin), and lignin, which are suitable for biofuel production. Various kinds of thermochemistry and biochemistry, as well as their combined processes, are used to recover bioenergy from PP [16,17].

Among many treatment technologies of PP, HTC is considered to be an environmentally friendly thermochemical conversion technology, which mainly converts PP into hydrochar under autogenous pressure (2–10 MPa) at 150–250 °C in a closed reactor (autoclave). The PP-derived hydrochar can be used in many fields directly or after proper activation or modification. The Co-HTC of PP and polyvinyl chloride produced hydrochar with enhanced fuel properties and dichlorination [18]. Ma et al. [19] and Xue et al. [20] found that the blends of hydrochar obtained from the HTC of PP and coal yielded enhanced combustibility and reduced CO_2_ emission. In addition, PP-derived hydrochar was usually utilized as a precursor and successfully converted into functional materials, such as hydrogels [21], aerogels [22], adsorbents [23], carbon nanosheets [24], etc.

The HTC process can be carried out by using both conventional electric heating and microwave heating. The combination of hydrothermal treatment and microwave heating could make the thermal treatment greener, faster, and more efficient [25]. In recent years, microwave-assisted HTC has been applied to the treatment of various biomasses, including lignocellulose [26,27,28], algae [29,30,31], animal manure [32,33], and sewage sludge [34]. PP is a kind of typical lignocellulose, which has its special structural characteristics, that is, a thick foam structure. It is very interesting to further clarify the characteristics and application potential of hydrochar produced by microwave-assisted HTC of PP, which may provide a new theoretical basis for the application of hydrochar derived from PP. So far, at present, there are few reports about the microwave-assisted HTC of PP. Semerciöz et al. [35] tried to use microwave-assisted HTC technology to treat PP and evaluated the adsorption performance of hydrochar. However, this study was only carried out at a fixed reaction temperature (213 ± 2 °C), lacking the evaluation of the yield change and other properties of hydrochar derived from PP under microwave-assisted HTC.

In this study, the microwave-assisted HTC of PP was carried out at a series of reaction temperatures (130–230 °C) under non-catalytic and CaO-catalytic conditions for the first time. Firstly, the changes in the yields of hydrochar obtained at various conditions were investigated. Then, the elemental and proximate compositions, thermal stability, combustion characteristics, and surface properties of representative hydrochar were assessed, in the hope of determining the application potential of PP-derived hydrochar. Lastly, the basic physicochemical characteristics of process water were also evaluated.

## 2. Materials and Methods

### 2.1. Materials

PP was provided by the science and technology academy of Jing-gang pomelo, Jiangxi Province, specializing in the research and promotion of pomelo planting and the corresponding product-processing technology. Fresh PP was sliced first and then naturally dried with the help of sunlight. To accurately control the solid–liquid ratio in the HTC process, the naturally dried PP was further put into the oven for drying treatment (drying at 105 °C for 12 h). The dried PP was then crushed and sieved, and the particles among 40–60 mesh were collected. The PP powder was put in a self-sealing bag and stored in a dryer for use. Ultra-pure water with a resistivity of about 18.0 MΩ·cm was used as a solvent in the microwave-assisted HTC process. In addition, the catalyst calcium oxide was purchased from Xilong Technology Co., Ltd. (Shenzhen, China) and belonged to an analytical reagent with 99.9% purity.

### 2.2. Microwave-Assisted HTC Experiments

The microwave-assisted HTC experiments of PP were realized by a microwave chemical synthesis instrument (XH-8000Plus), which was manufactured by Xianghu Technology Co., Ltd. (Beijing, China). The maximum design reaction temperature and pressure are 260.0 °C and 6.0 Mpa, respectively. Five different reaction temperatures were involved (150/170/190/210/230 °C) and the reaction time and solid–liquid ratio were fixed at 60 min and 0.025 g/mL, respectively. In a typical microwave-assisted HTC experiment, about 1.0 g of PP was mixed with 40 mL of ultra-pure water and transferred to a closed reactor (effective working volume of 100 mL). If the catalyst was involved, the amount of catalyst was fixed at 10.0% of the mass of raw material. The sealed reactor was then placed in the oven and heated to the desired reaction temperature, which would be maintained for the required time. After this, the reactor was cooled down to room temperature with the aid of a built-in fan. At last, the solid/liquid mixture was separated through vacuum filtration with the aid of a Whatman quantitative filter paper. The obtained solid product was then put into an oven for drying at 105 °C for 12 h, and the dried solid product was defined as the hydrochar (HC) (including solid residue components) and its yield (*Y*_1_, wt.%), calculated according to Equation (1). The obtained liquid product was defined as process water (PW).

(1) $$Y_{1}=\frac{M_{HC}}{M_{PP}+M_{catalyst}}\times 100$$

where *M_PP_*, *M_HC_*, and *M_catalyst_* were the mass of the raw PP feedstock, HC product, and catalyst, respectively.

### 2.3. Analysis of Raw PP and HC

#### 2.3.1. Proximate and Elemental Analysis

A muffle furnace (SX2-2.5-10NP, Yiheng, Shanghai, China) was used to determine the content of volatile matter (VM) and ash in raw PP and HC products in light of the methods reported in previous literature [36]. The content of fixed carbon (FC) was calculated by Equation (2).

(2) $$C_{FC}=100\%-C_{VM}-C_{Ash}$$

where *C_FC_*, *C_VM_*, and *C_Ash_* were the percentages (wt.%) of the fixed carbon, volatile matter, and ash in raw PP feedstock and HC products on a dry basis, respectively.

The percentages of carbon (C), hydrogen (H), and nitrogen (N) in raw PP and its derived HC products were analyzed with the aid of an elemental analyzer (EL III, Elementar, Hanau, Germany). The corresponding percentage of oxygen (O) was estimated by the difference method. Meanwhile, the caloric values (higher heating value (*HHV*), MJ/kg) of raw PP and its derived HC products were calculated according to Equation (3), based on the elemental compositions [37,38].

(3) $$HHV=0.382\times P_{C}+0.849\times \left(P_{H}-\frac{P_{O}}{8}\right)$$

where *P_C_*, *P_H_*, and *P_O_* were the percentages (wt.%) of the carbon, hydrogen, and oxygen in raw PP feedstock and HC products on a dry basis, respectively.

To assess the recovery of energy during the microwave-assisted HTC process, energy densification (*ED*, an important fuel property) was defined as the ratio of the calorific value of HC to that of raw PP feedstock (Equation (4)).

(4) $$ED=\frac{HHV_{HC}}{HHV_{PP}}$$

where *HHV_HC_* and *HHV_PP_* were the calorific values (MJ/kg) of HC products and raw PP feedstock, respectively.

#### 2.3.2. Analysis of pH, EC, and Nutrients

To determine the pH and electrical conductivity (EC) of the raw PP feedstock and HC products, the leaching solutions of PP and HC products were prepared according to the method reported in previous literature [39]. According to the liquid–solid ratio of 1:10 (*w*/*v*), a certain amount of PP or HC sample was fully mixed with ultra-pure water, and the supernatant was obtained by centrifugation after reciprocating oscillation for 4 h at room temperature. Then, the pH and EC of the supernatant were detected with the aid of a pH meter (HS-3C, INESA, Shanghai, China) and a conductivity meter (DDS-11A, INESA, Shanghai, China), respectively. Both the total and soluble contents of main nutrients (N/P/K) in raw PP and HC products were determined to evaluate the nutrient changes. To detect the total phosphorus (TP), the raw PP and HC products were melted with NaOH. With regard to total potassium (TK), the raw PP and HC products were digested with HNO_3_-H_2_O_2_ (30%)-HCl. The soluble phosphorus (SP), soluble potassium (SK), and soluble nitrogen (SN, including NO_3_-N and NH_4_-N) were extracted with NaHCO_3_ (0.5 mol/L), NH_4_OAC (1.0 mol/L), and KCl (1 mol/L), respectively. The detailed details of the above measurements were described in the literature [34,40]. The total nitrogen (TN) was estimated according to the elemental analysis results.

#### 2.3.3. Thermogravimetric Analysis

The thermogravimetric analysis of the raw PP and its derived HC products was conducted with the aid of a thermogravimetric analyzer (Discovery TGA55, TA Instruments-Waters, Newcastle, USA). A certain amount of PP or HC (about 10.0 mg) was put on a platinum plate, and then the sample was heated from room temperature (30 °C) to 900 °C at a constant heating rate (20 °C/min). Nitrogen and air were used as the carrier gas, and the flow of carrier gas was set at 100 mL/min. The pyrolysis and combustion characteristics of PP or HC were evaluated according to the TG-DTG (thermogravimetry–derivative thermogravimetry) curves obtained in nitrogen and air, respectively.

To accurately assess the pyrolysis performance of raw PP and HC, the devolatilization index (*DI*) was introduced and *DI* was defined in Equation (5) [41].

(5) $$DI=\frac{DTG_{max}}{T_{in}\times T_{m}\times \Delta T_{1/2}}$$

where *DTG_max_* is the maximum weight loss rate (wt.%/min); *T_in_* is the initial devolatilization temperature (°C), corresponding to a weight loss of 5%, concerning the final weight loss; *T_m_* (°C) is the temperature corresponding to *DTG_max_*; and ∆*T*_1/2_ is the temperature interval when *DTG*/*DTG_max_* equals 1/2 (°C).

To accurately assess the combustion performance of raw PP and HC, two important indexes were calculated according to Equations (6) and (7), i.e., comprehensive combustion index (*CCI*) and combustion stability index (*CSI*).

(6) $$CCI=\frac{DTG_{max}\times DTG_{mean}}{T_{i}^{2}\times T_{b}}$$

(7) $$CSI=\frac{DTG_{max}}{T_{i}\times T_{m}}\times 8.5875\times 10^{7}$$

where *DTG_mean_* is the average weight loss rate (wt.%/min), *T_i_* is the ignition temperature (°C), *T_b_* is the burnout temperature (°C), *DTG_max_* is the maximum weight loss rate (wt.%/min), and *T_m_* (°C) is the temperature corresponding to *DTG_max_*. 

#### 2.3.4. Surface/Pore Structure Analysis

Scanning electron microscopy (SEM, Regulus 8100, Hitachi, Tokyo, Japan) was applied to observe the surface morphologies of the raw PP and its derived HC products, which were pre-coated with gold. The surface functional groups of the raw PP and its derived HC products were observed through an intelligent Fourier transform infrared spectrometer (Nicolet5700, Thermo Nicolet, Madison, WI, USA) in transmission mode with a spectral range of 4000–400 cm^−1^ (4 cm^−1^ resolution, 32 times scans). To determine the pore structure of hydrochar, nitrogen adsorption–desorption experiments were performed with the aid of a specific surface and porosity tester (JW-BK132F, JWGB, Beijing, China).

### 2.4. Analysis of PW

With the aid of a pH meter (HS-3C, INESA, Shanghai, China) and a conductivity meter (DDS-11A, INESA, Shanghai, China), the pH and EC values of PW were detected. The content of organic matter in PW was also estimated through the chemical oxygen demand index (COD), which was determined according to the potassium dichromate oxidation method. The contents of soluble phosphorus (SP) and soluble potassium (SK) in PW were analyzed by the colorimetric method and flame photometer method, respectively. Nash’s reagent colorimetry was applied to determine the content of NH_4_^+^-N in the PW [34].

## 3. Results and Discussion

### 3.1. Yields of HC

**Figure 1:**
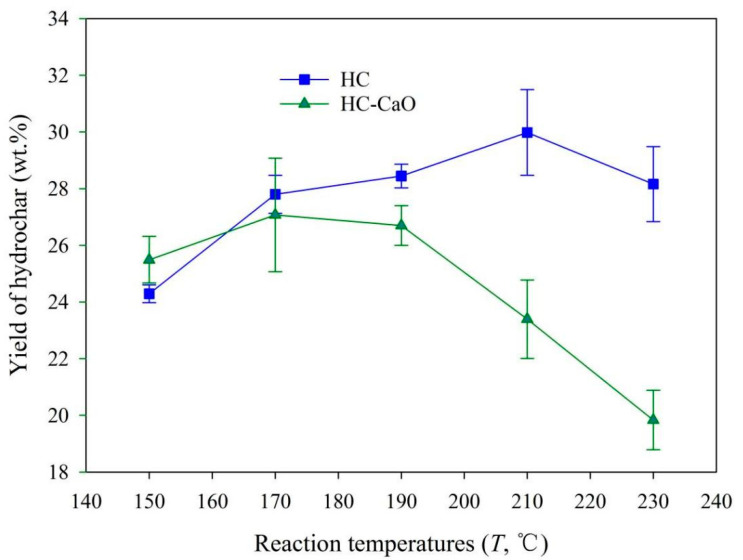
Yield of HC products obtained from the microwave-assisted HTC of PP.

The yields of HC obtained from the microwave-assisted HTC of PP at different reaction temperatures (150–230 °C) with or without CaO as the catalyst are depicted in Figure 1. As shown in Figure 1, the increase in reaction temperature initially promoted the information of HC in the process of non-catalytic HTC. For example, the yield of HC obtained under non-catalytical conditions was elevated from 24.3 ± 0.3 wt.% at 150 °C to 30.0 ± 1.5 wt.% at 210 °C. When the reaction temperature exceeded 210 °C, the yield of HC was almost unchanged. In general, the reaction rate in the HTC process was influenced by reaction temperature to a substantial degree, now that the reaction temperature initiated an ionic reaction (i.e., the hydrolysis reaction) in the sub-critical regions. Higher temperatures facilitated dehydration, decarboxylation, and condensation reactions at the same time. The dissolution of the intermediary was improved, thus promoting consequent transformation via polymerization and secondary char formation at higher temperatures [42].

Interestingly, when CaO was introduced as a catalyst, the changing trend of hydrochar yield was quite different. At the initial stage of reaction temperature rise (150–190 °C), the yield of hydrochar was relatively stable. When the reaction temperature exceeded 190 °C, the yield of HC was reduced with further increases in reaction temperature. For example, the yield of HC obtained in the catalytical HTC process was 26.7 ± 0.7 wt.% at 190 °C and it was reduced to 19.8 ± 1.1 wt.% at 230 °C. The reason for the above results may be that the secondary disintegration and decomposition reactions of solid residues (hydrochar) were enhanced with the continuous rise of reaction temperature, resulting in promoting the information of the constituents of liquid or incondensable low gas molecules (Table S1) [42,43].

Calcium oxide is a commonly used conditioner in the process of soil remediation and improvement. Using CaO as a catalyst does not need to consider the recovery and toxicity of the catalyst. In the HTC process of pig manure and sewage sludge, CaO has been proven to be a good catalyst, which not only improved the yield of HC but also reduced the harmful components (such as heavy metals and polycyclic aromatic hydrocarbons) in HC to a certain extent [33,34,44,45,46]. Interestingly, the addition of CaO in the microwave-assisted HTC of PP as the catalyst inhibited the information of HC on the whole. Nizamuddin et al. [47] proposed that, in general, in the HTC of biomass, alkaline catalysts will inhibit the formation of solid-phase products, which, in turn, will promote the formation of liquid-phase products. The main reasons for the above differential influence results may be that PP is typical lignocellulosic biomass (mainly consisting of lignin, cellulose, and hemicellulose and possessing low inorganic component content, as shown in Table 1), while pig manure and sewage sludge are typical heterogeneous mixtures (mainly consisting of protein, lipid, and carbohydrate and generally containing high inorganic component content). In the process of the HTC of pig manure and sewage sludge with high ash content, the introduction of CaO may promote the formation of insoluble inorganic salts, thus ultimately improving the yield of solid-phase products [34,44]. Special attention needs to be paid to the fact that, in addition to the catalytic effect of CaO itself, the use of CaO as a catalyst will change the pH value of the HTC reaction system, which, in turn, will have a certain impact on the HTC of biomass. In the future, more in-depth related research should further clarify the specific mechanism of CaO affecting the HTC of biomass.

### 3.2. Proximate/Elemental Compositions of HC

Table 1 presents the proximate/elemental compositions of raw PP and its derived HC products. The raw PP contained high contents of volatile organic components (97.5 wt.%) and low contents of ash (2.0 wt.%) and fixed carbon (0.5 wt.%). Usually, volatiles mainly exist in organic grease substances and oxygen-containing compounds. In the HTC process, most of the volatiles were degraded and transformed into HC, aqueous components (existing in PW), and gases. Meanwhile, some organic grease substances and oxygen-containing compounds were converted into fixed carbon and inorganic salts due to the partial decomposition of volatiles [19]. It was also reported that the HTC process had desalting effects [31,48,49]. Thus, it was found that the HC obtained at 210 °C without catalyst (HC-210) contained lower content of ash compared to the raw PP (0.1 wt.% versus 2.0 wt.%). Similar results can be found in the research of Liang et al. [49], Radojčin et al. [50], and Mendoza Martinez et al. [51]. The increase in ash content in the HC produced at 190 °C with CaO as the catalyst (HC-CaO-190) may be ascribed to the formation of some insoluble calcium salts.

**Figure 2:**
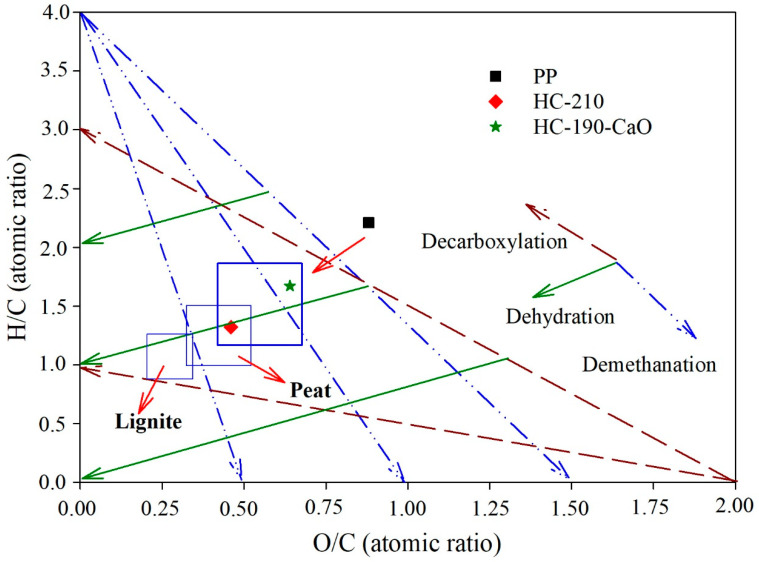
Van Krevelen diagram for raw PP and HC products.

Compared to the raw PP, the contents of carbon in HC products were increased, while that of hydrogen and oxygen was reduced, resulting in a decrease in the molar ratios of H/C and O/C. Dehydration and decarboxylation reactions contributed to the decrease of H and O in HC products, while the increase in C may be mainly due to the condensation and aromatization reactions that occurred during the microwave-assisted HTC process [20]. As depicted in Figure 2, a greater decrease was observed in the molar ratio of H/C than that of O/C, indicating that the dehydration reaction, involving the elimination of hydroxyl groups (formation of water) played a more important role than the decarboxylation reaction, associated with the thermal cleavage of long-chain carboxylic acids (formation of carbonyls, carboxylic acid, and CO_2_) [52,53]. The H/C, O/C, and (N+O)/C molar ratios have been widely used to estimate the aromaticity, carbonization degree, and polarity of materials, respectively [36]. The HC products had lower H/C, O/C, and (N+O)/C molar ratios, meaning lower polarity and higher aromaticity and carbonization degree. The raw PP feedstock had a caloric value of 17.4 MJ/kg, while the HC products possessed higher caloric values of 19.8–23.8 MJ/kg in consideration of the increase in aromaticity and carbonization degree, which was comparable to the caloric value of lignite (22.2 MJ/kg) [20]. Accordingly, the energy densification reached 1.1–1.4, meaning that the energy was more intense in PP-derived HC products. The contents of nitrogen in raw PP and HC products were both relatively low, leading to a high C/N mass ratio (52.5–61.5). A high C/N mass ratio (exceeding 20) would not ensure sufficient available nitrogen for plant utilization [54].

### 3.3. The pH, EC, and Nutrient Compositions of HC

Table 2 presents the pH, EC, and nutrient compositions of PP feedstock and HC products. The HC obtained at 210 °C (HC-210) presented a similar pH value as that of PP feedstock, approximately 5.0. Unlike pyrolysis biochar (which is usually alkaline), hydrochar is often acidic, which may be due to the formation of insoluble salts, the dissolution of alkaline components in the PW, and the rich acidic groups in HC [39]. However, with the addition of CaO as the catalyst, the pH value of PP-derived HC was up to 6.2, which might be due to the acid-neutralizing capacity of CaO. The near-neutral characteristics of HC-190-CaO were also more conducive to its use in land use or improvement. After the HTC treatment, partial salts in raw feedstock will be transferred into PW. Thus, it was found that HC-210 showed a lower EC value (0.5 ms/cm) than that of PP feedstock (1.0 ms/cm). In the case of HC-190-CaO, the EC value reached up to 1.9 ms/cm.

The HTC treatment also had a significant effect on the changes in nutrients (N/P/K). Firstly, the total content of nitrogen (TN) was improved in HC products, but the soluble nitrogen (SN) was reduced. The polymerization of the hydrolysates of aldehyde and amino groups through the Maillard reaction increased TN. The decrease in SN may be attributed to the loss of NH_3_ during HTC reactions [39]. After the HTC treatment, the total phosphorus (TP) in HC-210 was slightly reduced compared to the PP feedstock, while it was increased to 2.6 g/kg in HC-190-CaO. However, the contents of soluble phosphorus (SP) in hydrochar were both decreased. P in biomass is mostly bound in phospholipids, nucleic acids, and protein. During the microwave-assisted HTC treatment, P was firstly dissociated from organic tissue, and then part of the soluble phosphates would be transformed into insoluble forms and thereafter deposited on the HC surface. The addition of calcium oxide catalyst promoted the formation of insoluble phosphorus-containing components [55]. The total potassium (TK) and soluble potassium (SK) in HC products were both reduced in a great measure compared to that in the PP feedstock, from 15.8 g/kg and 13.2 g/kg to 1.0–2.1 g/kg and 1.0–1.3 g/kg, respectively. It was speculated that most of the dissociated K were distributed in the PW, while a little part was retained on the surface of the HC.

### 3.4. Surface and Pore Structure Characteristics of HC

#### 3.4.1. Surface Morphology of HC

**Figure 3:**
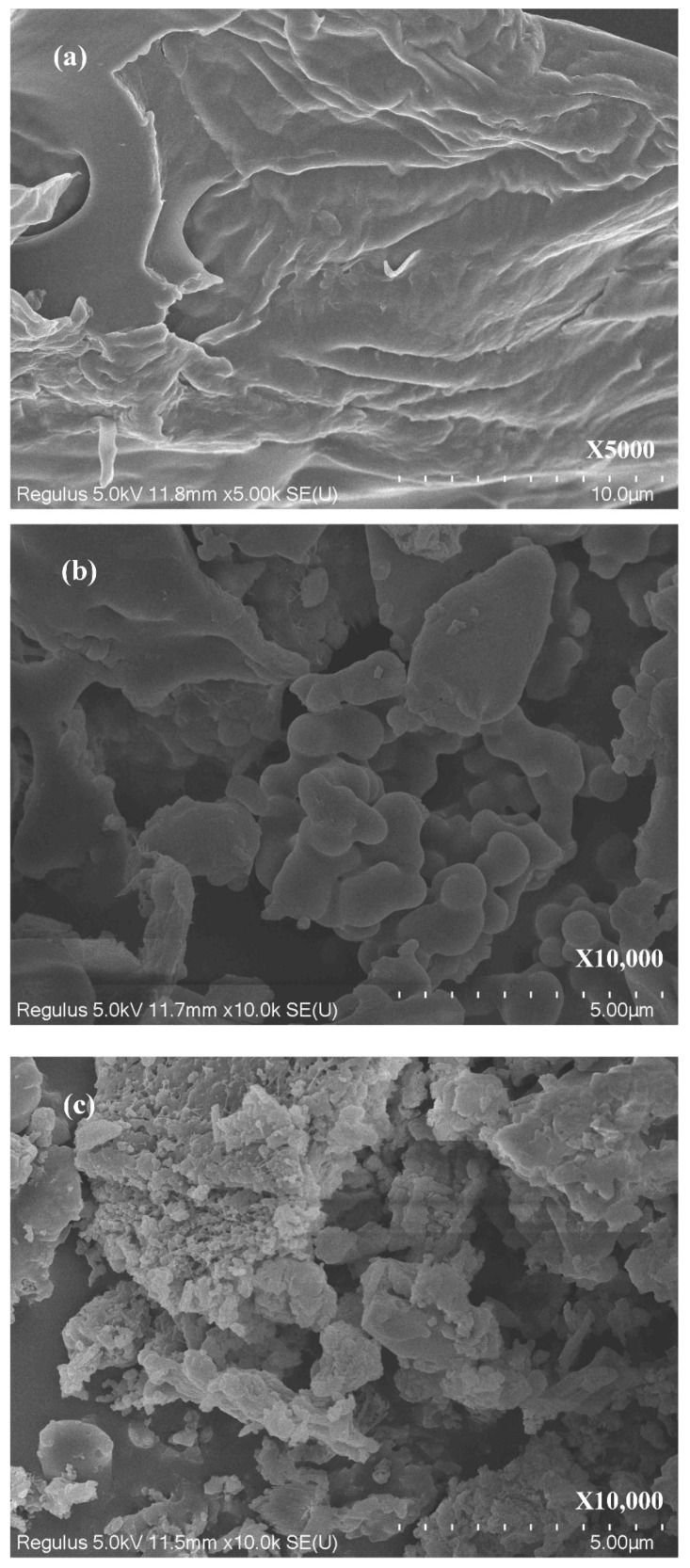
SEM images of PP and HC products. (**a**) raw PP, (**b**) HC obtained at 210 °C without catalyst, (**c**) HC obtained at 190 °C with CaO as the catalyst.

The surface morphology evolution of PP during the microwave-assisted HTC process was characterized by the SEM images, and the results are presented in Figure 3. As shown in Figure 3a, the raw PP feedstock presented a dense and smooth surface structure (unbroken plane), and the pores in the raw PP feedstock appeared to be clogged. After the HTC treatment, the dehydration and devolatilization reactions eventually opened these clogged pores. Spaces, opened slits, and spherical shape bulge forms were present on the surface of HC products. In particular, the PP-derived HC without a catalyst possessed a layered structure with debris accumulation (Figure 3b). In the presence of CaO as the catalyst, the surface of HC became coarse with many small pores (Figure 3c). These pores were mainly created by the escape of volatiles during the microwave-assisted HTC process [31,49,56].

#### 3.4.2. Surface Functional Group of HC

**Figure 4:**
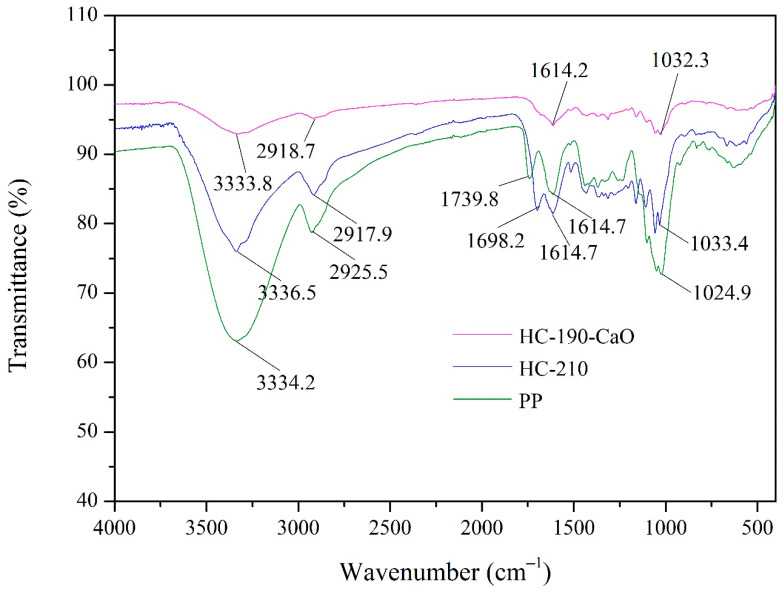
FTIR spectra of PP and HC products.

The changes in functional groups of HC products in comparison with the raw PP are shown in Figure 4. The characteristic peaks of PP observed at 3334 cm^−1^ and 2926 cm^−1^ represented the -OH stretching in hydroxyl groups and -C-H stretching in methyl, methylene, and methyne groups, respectively [19]. Both of the above two peaks in HC products were significantly weakened. Peaks at 1740 cm^−1^ and 1615 cm^−1^ of PP belonged to C=O stretching in carbonyls and C=C stretching in the benzene ring (lignin), respectively [23]. In HC products, the characteristic peak of C=C groups was weakened, meaning that some lignin fragments and intermediate structures remained in hydrochars. In the case of C=O groups, the corresponding characteristic peak disappeared in HC products because of the starting of possible hemicellulose decomposition, while in HC-210, a new peak at 1698 cm^−1^ was formed, indicating C=O bonding in ketone, aldehyde, and carboxylic acid functional groups. Peaks at around 1160 cm^−1^ of both HC products can be attributed to the C−O−C stretching of glycosidic bonds of cellulose [35]. The characteristic peak of C−O bending in cellulose was observed at 1025–1051 cm^−1^, which was significantly weakened in both the HC products, suggesting the partial degradation of cellulose during the HTC reaction [19,20].

#### 3.4.3. Pore Structure of HC

Table 3 lists the specific surface area, pore size, and pore volume of PP and HC products. The raw PP feedstock has a low specific surface area of 0.7 m^2^/g. After the microwave-assisted HTC treatment, the specific surface area was increased to 3.7 m^2^/g for HC-210 and 4.3 m^2^/g for HC-190-CaO. Meanwhile, the average pore size and pore volume were both promoted, suggesting that the microwave-assisted HTC treatment developed the pore structure of HC. The pores in materials usually include three types: micropores (<2 nm), mesopores (2–50 nm), and macropores (>50 nm) [38,57,58]. Thus, according to the average pore size, the raw PP and its derived HC products all belonged to mesopore materials. In general, the specific surface area of PP-derived HC products is relatively small. If it is to be used as an adsorbent, it should be properly activated [35].

**Figure 5:**
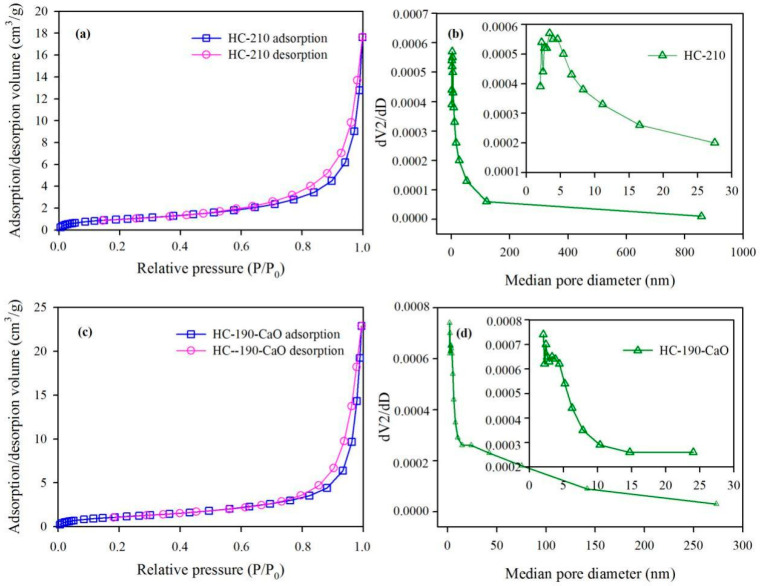
N_2_ adsorption–desorption isotherms and pore size distribution of HC products. HC-210: (**a**) and (**b**); HC-190-CaO: (**c**) and (**d**).

The nitrogen adsorption–desorption isotherms and pore size distribution of HCs are shown in Figure 5. It can be seen in Figure 5a,c that when the relative pressure (P/P_0_) was higher than 0.9, the adsorption/desorption volume showed a sharp increasing trend, and the hysteresis loop appeared at the high-pressure end. According to the classification of adsorption isotherms reported by Sing et al. [59], both HC products belonged to the Type IV isotherm, usually given by many mesoporous industrial adsorbents. Furthermore, the hysteresis loop can be classified into the Type H3 loop, usually observed with aggregates of plate-like particles giving rise to slit-shaped pores. The distribution curves of pore size for hydrochars showed a similar peak horizontal position (about 3.0 nm). The HC-210 presented a wide range of pore size distribution, and the percentage of pores (>200 nm) reached 24.2%. Meanwhile, the percentage of pores (51–200 nm) in HC-190-CaO was up to 45.6%.

### 3.5. Pyrolysis and Combustion Characteristics of HC

#### 3.5.1. Pyrolysis Characteristics of HC

**Figure 6:**
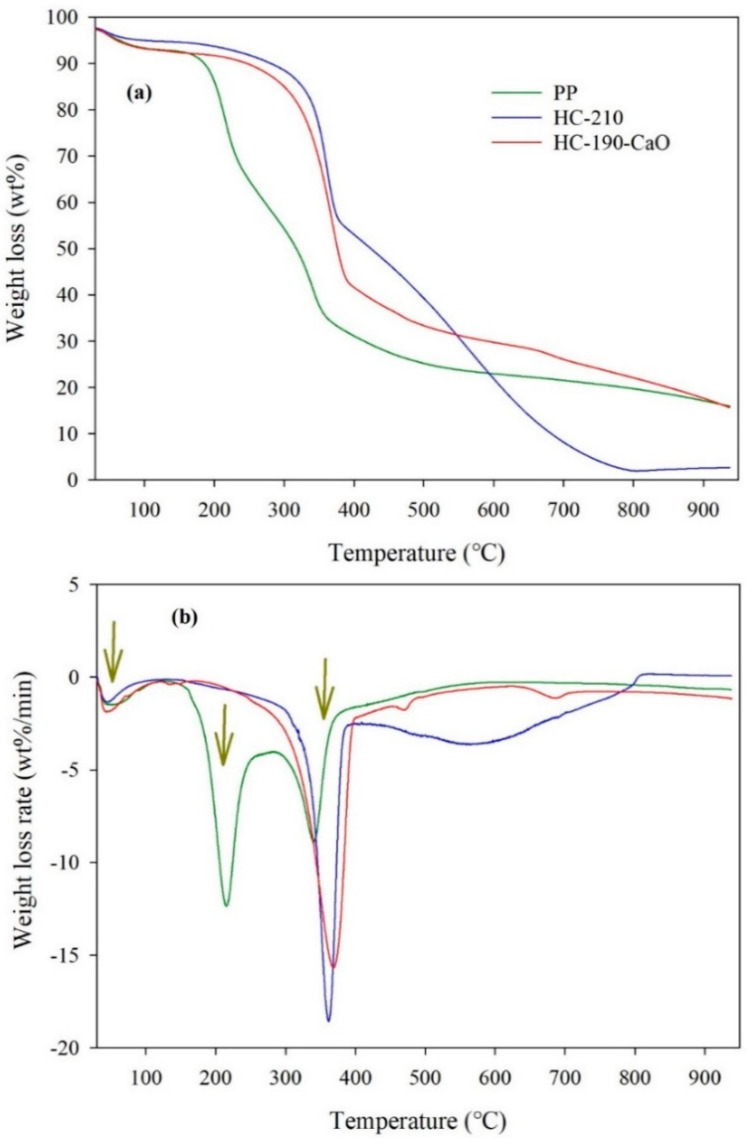
TG-DTG curves of PP and its derived HC products under nitrogen conditions. TG (**a**) and (**b**) DTG.

The pyrolysis process of raw PP and HC products was simulated through the thermogravimetric analysis under nitrogen conditions (Figure 6). The corresponding pyrolysis parameters were listed in Table 4. In the case of raw PP, the pyrolysis process included three stages. The first stage (under 150 °C) was mainly involved in the water evaporation (as seen in the first degradation peak in Figure 6b for raw PP) and was next followed by the decomposition of hemicellulose, cellulose, and lignin (150–500 °C, the second stage). The last stage (higher than 500 °C) was mainly related to the degradation of residual volatile matter, fixed carbon, and inorganic matter. During the second stage, the DTG curve of raw PP had two degradation peaks, i.e., 215.4 °C and 340.4 °C. The first peak represented the decomposition peak of hemicellulose, considering that the decomposition temperature range of hemicellulose is 150–350 °C [56]. Cellulose had a little greater decomposition temperature demand than hemicellulose, which was constituted of polymers with a long chain of glucose without branches. Lignin was decomposed in a wider temperature range of 160–500 °C. Thus, the second peak was ascribed to the decomposition peak of cellulose and lignin [60].

Notably, the first peak in the second stage disappeared for the PP-derived HC products, which suggests that most of the hemicellulose has been degraded/converted during the HTC treatment and the structure of PP-derived HC products had higher thermal stability [19,49]. Meanwhile, for the DTG curves of HC products, the second degradation peak in the second stage was intensified, with the peak mass loss rate increasing from 8.9 wt.%/min (raw PP) to 15.7 wt.%/min (HC-210) and 18.6 wt.%/min (HC-190-CaO), respectively. It can be inferred that the HC products possessed higher devolatilization ability and the HTC treatment improved the degradability of residual lignin. That can be further proved by the increased devolatilization index (*DI*, from 2.3 (PP) to 5.7–7.6 (hydrochars), Table 4). In general, higher *DI* suggested the easier release of volatile matters, which was beneficial for fuel.

#### 3.5.2. Combustion Characteristics of HC

**Figure 7:**
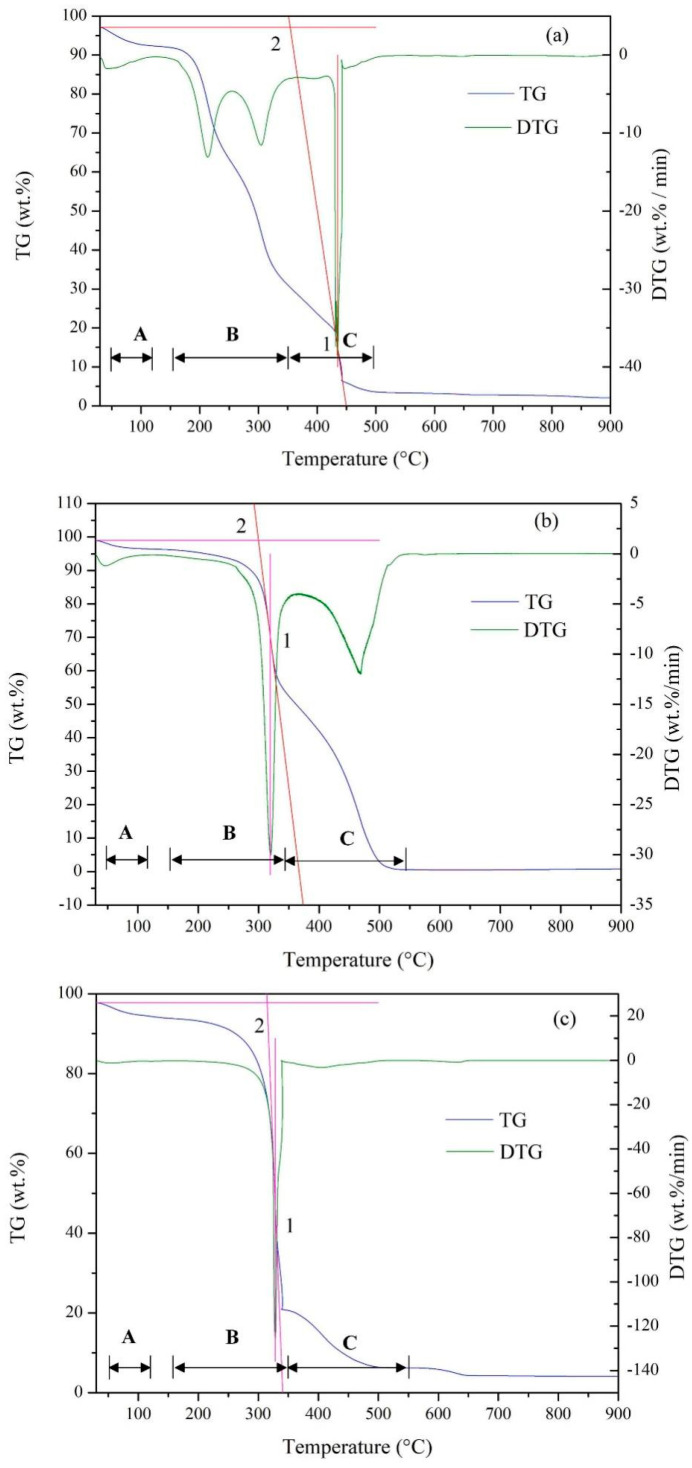
TG-DTG curves of PP and its derived HC products under air conditions. (**a**) PP, (**b**) HC-210, and (**c**) HC-190-CaO.

The combustion process of raw PP and HC products was simulated through the thermogravimetric analysis under air conditions (Figure 7). The corresponding combustion parameters were listed in Table 5. In the case of raw PP (Figure 7a), the combustion process included three stages. Stage A (under 120 °C) was mainly attributed to water evaporation. The combustion of cellulose and hemicellulose dominated in Stage B, ranging from 150–350 °C. The combustion of lignin and fixed carbon were responsible for Stage C, ranging from 350–500 °C. After the above-mentioned three stages, the dominant process was the degradation of inorganic substances. As shown in Figure 7b, the main combustion process of HC-210 was Stage B, which was mainly the combustion of hydrochar, followed by Stage C, the combustion of residual lignin and fixed carbon. The addition of CaO as a catalyst further promoted the combustion process, the *DTG_max_* of Stage B reached up to 125.9 wt.%/min, and Stage C was relatively unapparent (Figure 7c).

The HC products possessed lower ignition temperatures (*T_i_*, 299.8–314.9 °C) compared to the raw PP (354.1 °C) but higher burnout temperatures (*T_b_*, 479.9–519.1 °C versus 473.2 °C), suggesting that the PP-derived HC products had better ignition performance and longer combustion time [60]. Accordingly, the PP-derived HC products presented better combustion performance and stability in comparison to raw PP, as proved by the increase in *CCI* and *CSI* indexes (Table 5). The improvement of combustion property was mainly due to the lower H/C and O/C ratios and more porous structure in the produced HC products. The PP-derived HC products possessed lower H/C and O/C ratios (Table 1), which made the HC products consume less energy during combustion and less soot was generated [18,56]. As shown in Table 3 and Figure 3, an obvious porous structure developed on the surface of HC products, which increased the contact with O_2_ during the combustion process [19]. Hence, the overall combustion efficiency of HC products was enhanced. Meanwhile, it was found that the HC products obtained with CaO as a catalyst resulted in higher mass residue after combustion compared to the raw PP. The high mass residue was consistent with the higher content of ash in HC-190-CaO. The better combustion property of HC-190-CaO may also be related to the higher content of fixed carbon and lower content of volatile matters compared to the raw PP. As reported, the higher contents of volatile matter were beneficial in maintaining a lower ignition point of the fuel, but the fixed carbon is also important for the combustion reaction because it determines the combustion time and energy of the fuel [19,60].

### 3.6. Characteristics of PW

The key properties of PW are listed in Table 6. The pH values of PW-210 and PW-190-CaO were 3.7 and 5.0, respectively, suggesting their acid property. The alkaline characteristics of the CaO catalyst resulted in the increase in the pH value of PW. The acid property of PW was mainly due to the presence of various organic acids produced during the microwave-assisted HTC of PP [26,53]. The EC of PW was about 1.4–3.3 ms/cm, suggesting that during the microwave-assisted HTC treatment, some soluble salts were migrated into PW [53]. A significant amount of NH_4_^+^N (592.2–939.6 mg/L), soluble K (379.4–353.9 mg/L), and soluble P (2.3–14.4 mg/L) were found in PW, which implies that the spent liquor may have a high potential for nutrient recovery. Meanwhile, high content of organic matter in PW was observed with a COD value of 3841.7–4991.7 mg/L, consisting of saccharides, organic acids, and some furans and phenolic compounds [39]. In general, PW cannot be discharged directly; otherwise, it will cause water environmental pollution. Now, researchers have explored many resource recycling technologies for the PW obtained from the HTC of biomass, such as anaerobic digestion, valuable chemical separation, algal cultivation, bioelectrochemical systems, and recirculation as an HTC solvent, etc. [61,62].

## 4. Conclusions

Microwave-assisted HTC technology was applied to treat PP in this study. The findings indicated that PP can be converted into multifunctional materials through microwave-assisted HTC treatment. PP-derived HC products possessed higher caloric value and better combustion properties, facilitating the use of solid fuel. PP-derived HCs presented higher porosity and more stable nutrients, facilitating land use. The pH value of HC and the content of phosphorus in HC were promoted when CaO was applied as the catalyst. The PW still contained a high content of organic matter and rich nutrient elements, which needed to be further recycled. In the research of microwave-assisted HTC of PP, the reaction mechanism, application of hydrochar, and analysis of aqueous products should be further improved based on this preliminary study in the future.

## Figures and Tables

**Table 1:** Proximate/elemental compositions of raw PP and HC products.

Items	Elemental Compositions (wt.%) ^a^				H/C ^c^	O/C ^c^	(N+O)/C ^c^	C/N ^d^	Proximate Compositions (wt.%) ^a^			HHV (MJ/kg)	ED
	C	H	N	O ^b^					Ash	VOM	FC ^b^		
PP	41.2	7.6	0. 7	48.6	2.2	0.9	0.9	61.5	2.0	97.5	0.5	17.4	-
HC-210 ^e^	57.3	6.3	1.1	35.3	1.3	0.5	0.5	54.0	0.1	99.8	0.1	23.8	1.4
HC-190-CaO ^f^	47.7	6.7	0.9	41.0	1.7	0.6	0.7	52.5	3.7	95.5	0.8	19.8	1.1

^a^ On a dry basis; ^b^ By difference; ^c^ Molar ratio; ^d^ Non-molar ratio; ^e^ Obtained at 210 °C; ^f^ Obtained at 190 °C with CaO as the catalyst.

**Table 2:** The pH, EC, and main nutrients of raw PP and HC products.

Items	pH	EC (ms/cm)	Main Nutrients (g/kg)					
			TP	SP	TK	SK	TN ^a^	SN
PP	4.9	1.0	1.3	0.6	15.8	13.2	6.7	0.8
HC-210	5.0	0.5	0.6	0.03	1.0	1.0	10.6	0.5
HC-190-CaO	6.2	1.9	2.6	0.4	2.1	1.3	9.1	0.6

^a^ Calculated from the elemental analysis results.

**Table 3:** Specific surface area, pore size, and pore volume of raw PP and HC products.

Items	Specific Surface Area (m^2^/g) ^a^	Average Pore Size (nm) ^b^	Micropore Volume (cm^3^/g) ^c^	Pore Volume (cm^3^/g) ^b^	Pore size Distribution (%) ^b^					
					≤2.0 nm	3–10 nm	11–50 nm	51–100 nm	101–200 nm	>200 nm
PP ^d^	0.7	9.6	- ^e^	0.002	-	-	-	-	-	-
HC-210	3.7	22.7	0.0014	0.028	0.1	14.7	34.0	17.3	9.7	24.2
HC-190-CaO	4.3	24.6	0.0015	0.036	0.2	11.5	33.8	24.6	21.0	8.9

^a^ BET (Brunauer–Emmett–Teller) multipoint method; ^b^ BJH (Barrett–Joyner–Halenda) method, adsorption data; ^c^ HK (Horvath and Kawazoe) method (pore size range: <2 nm); ^d^ Cited from the literature [19]; ^e^ Not determined.

**Table 4:** Pyrolysis parameters of raw PP and HC products.

Items	*T_in_* (°C)	*T_m_* (°C)	*DTG_mean_* (wt.%/min)	∆*T*_1/2_ (°C)	*DI* (wt.%·min^−1^·°C^−3^, ×10^−6^)
PP	161.3	215.4	12.4	155.6	2.3
HC-210	231.6	361.4	18.6	29.1	7.6
HC-190-CaO	146.0	368.8	15.7	51.0	5.7

**Table 5:** Combustion parameters of raw PP and HC products.

Items	PP	HC-210	HC–190-CaO
*T_i_* (°C)	354.1	299. 8	314.9
*T_b_* (°C)	473.2	519.1	479.9
*T_m_*	434.7	319.4	328.3
*DTG_max_* (wt.%/min)	38.8	30.1	125.9
*DTG_mean_* (wt.%/min)	2.2	2.2	2.1
*R_m_* (wt.%)	2.1	0.8	4.1
*CCI* (10^−6^, wt.%^2^·min^−2^·°C^−3^)	1.4	1.4	5.7
*CSI* (10^4^, wt.%·min^−1^·°C^−2^)	2.2	2.7	10.5

**Table 6:** Basic properties of PW (mg/L).

Items	COD	pH	EC (ms/cm)	SP	SK	NH_4_^+^-N
PW-210	3841.7	3.7	1.4	14.4	379.4	592.2
PW-190-CaO	4991.7	5.0	3.3	2.3	353.9	939.6

## Data Availability

Exclude this statement.

## Supplementary Materials

The following supporting information can be downloaded at: https://www.mdpi.com/article/10.3390/ma15249055/s1.
