# Supplementary material for: Recycling Pomelo Peel Waste in the Form of Hydrochar Obtained by Microwave-Assisted Hydrothermal Carbonization
Source: Materials (Basel). 2022 Dec 18;15(24):9055. doi: 10.3390/ma15249055 (PMC9782344; doi:10.3390/ma15249055)
Supplement: Supplementary file 1 [file materials-15-09055-s001.zip › materials-2060916-supplementary.pdf]

Recycling pomelo peel waste in the form of hydrochar obtained by microwave-assisted hydrothermal carbonization

Yu-jie Wang, Nan Li, Guo-rong Ni *, Chun-huo Zhou, Xin Yin and Hua-jun Huang *

School of Land Resources and Environment, Key Laboratory of Agricultural Resource and Ecology in the Poyang Lake Basin of Jiangxi Province, Jiangxi Agricultural University, Nanchang 330045, PR China

* Correspondence: Guo-rong Ni (ngr@jxau.edu.cn); Hua-jun Huang (huajunhuang@jxau.edu.cn), ORCID: 0000-0002-4104-9471

Table S1
Yields of gas and liquid products (wt.%)

Items		150	170	190	210	230
HC-210	Gas	5.33±0.58	6.33±0.58	7.33±1.53	10.67±1.15	16.66±1.52
	Liquid	70.38	65.87	64.22	59.35	55.18
HC-190-CaO	Gas	3.94±0.53	5.76±0.52	8.18±0.91	9.70±1.05	13.64±0.91
	Liquid	70.57	67.17	65.12	66.91	66.53

The yield of the gaseous product (Y_2, wt.%) was calculated according to Eq. (S1).

$$Y_2 = \frac{M_0 - M_1}{M_{PP} + M_{catalyst}} \times 100 \quad \text{(S1)}$$

where M_0 is the mass of the vessel and unconverted reactants inside it before the reaction; M_1 is the mass of the vessel and residual products inside it when gas escaped after the reaction; M_{PP} and $M_{catalyst}$ were the mass of the raw PP feedstock and catalyst, respectively.

The yield of liquid products (Y_3, wt.%) contained in process water was calculated by the interpolation method.

$$Y_3 = 100\% - Y_1 - Y_2 \quad \text{(S2)}$$
